# The Relationship of Creativity and Motor Creativity with Physical Activity and Motor Fitness in a Gender Perspective Among 8–9-Year-Old Children

**DOI:** 10.3390/children11121501

**Published:** 2024-12-10

**Authors:** Maryna Khorkova, Łukasz Bojkowski, Agata Korcz, Marlena Łopatka, Dagny Adamczak, Jana Krzysztoszek, Michał Bronikowski

**Affiliations:** 1 Department of Didactics of Physical Activity, Poznan University of Physical Education, 61-871 Poznan, Poland; korcz@awf.poznan.pl (A.K.); lopatka@awf.poznan.pl (M.Ł.); adamczakdagny@gmail.com (D.A.); krzysztoszek@awf.poznan.pl (J.K.); bronikowski@awf.poznan.pl (M.B.); 2 Department of Psychology, Poznan University of Physical Education, 61-871 Poznan, Poland; bojkowski@awf.poznan.pl

**Keywords:** early education children, motor creativity, fitness, sex differences, physical education

## Abstract

Background: In this study, we aimed to determine the presence of any potential gender differences and relationships in terms of creativity, physical activity (PA), motor fitness, and motor creativity in children aged 8–9 years. Methods: The study included 195 primary school children (92 girls and 103 boys) aged 8–9 years old from grade 2. To determine creativity, the Test for Creative Thinking-Drawing Production was used, while motor creativity was assessed using Torrance’s ‘Thinking Creatively in Action and Movement’ test. Motor fitness was assessed with the selected items from the Eurofit battery and Piórkowski tests. The level of moderate-to-vigorous PA was determined with the Physical Activity Screening Measure. The Mann–Whitney U test was employed for in-between group comparison and Spearman’s correlation to determine relationships between the variables. Results: The results of the research showed the presence of differences in motor fitness between boys and girls, but no differences in PA, creativity, as well as motor creativity between genders. Moreover, it was found there was no association between PA and motor fitness and creativity or motor creativity in either boys or girls at this age. Conclusions: Thus, it can be assumed that to develop creativity through PA in children aged 8–9 years, it might be necessary to create an appropriate environment and strategy that will strengthen, inspire, and promote children’s creativity through movement. Having established that there is no difference in creativity between genders at this age, it was pointed out that it is necessary to look for teaching methods that would effectively awaken this ability in a co-educational setting.

## 1. Introduction

Childhood is a special part of a human’s life, which for the most part determines further opportunities for personal development, the ability to reveal own potential in the future, and has a significant impact on one’s self-realization in adulthood [1]. Therefore, it is extremely important to create an environment in early childhood that promote the most effective conditions for development of a child, taking into account all influencing factors.

Movement is a fundamental aspect of development through the whole childhood. Children, born to be active and to discover, explore the world in different ways of moving [2], using movement as their first means of getting to know the world. One may even call it the first language in which an infant communicates their will. From the moment we are born, the brain gives priority to various forms of movement, and movement becomes one of the main functions in early life, after the basic survival functions of the heartbeat, breathing, and digestion. Every movement made by a child adds to the building of new important neural pathways in the brain and, thus, helps to develop their physical abilities [3]. The range of movements a child learns helps to develop basic (movement) skills that are necessary for the discovery of their immediate environment. It also helps them develop active and healthy bodies, which will have an impact on their quality of life in later years [2].

At the physical level, it has been shown that higher PA levels are associated with better body composition, higher bone mineral density, higher insulin sensitivity [4], maintenance of energy balance, and, consequently, healthy weight, healthy growth, development of the cardiorespiratory and musculoskeletal system, and avoidance of cardiovascular disease risk factors (e.g., hypertension, dyslipidemia, diabetes) [5,6].

Besides health improvement and disease prevention regular participation in PA is linked to enhancement of brain function and cognition. It also positively influences academic performance [7]. Several hypothesized mechanisms for explaining the process of how exercise can be beneficial for cognition have been proposed. This includes, among others, (1) increased blood and oxygen flow to the brain [8]; (2) increased levels of norepinephrine and endorphins [9,10], leading to a stress reduction and an improvement of mood [11]; and (3) increased growth factor, which helps to create new nerve cells and support synaptic plasticity [12,13].

The role of the physical activity, in general, and motor fitness, in particular on pediatric health, is certainly a topic of a great importance as we move toward a better understanding of the role that the physical, social, and cultural environments have on our population and their health. The association between cognition, brain structure and function, academic performance, and PA has been confirmed in many studies [6,14,15]. In a cross-sectional study by Donnelly et al. [4] it was indicated that the cohort-based studies involving PA have provided positive support for the relationship between PA and cognitive function. The authors pointed that it was the greater amounts or enhanced forms of PA that were associated with the improvements in cognitive functions. Acute PA studies also show a positive relationship between PA and cognition [4]. However, it should be noted the need for more in-depth research on this topic is required to be able to better establish causality and determine underlaying processes and potential long-term effects [4].

One of humans’ general cognitive skills is creativity, which has recently become an extremely important and in-demand skill in the challenging modern world. Creativity, which is one of the fundamental dispositions in everyday life, appears as a key concept of human innovation [16]. The traditional way of defining creativity is the ability to produce something that is both new/original and task/domain appropriate [17]. More recently, creativity is seen as in a multidimensional perspective that can be performed in art, science, commercial enterprise, and business innovation [18]. Baas et al. [19] define the roots of creative cognition in the arts and sciences and suggest that creativity is not just a cultural or social construct but that it is also psychological and cognitive process [18,20,21,22,23]. Despite this, many studies on creativity (including experimental ones) reported inconsistent findings [24,25,26]. One of the reasons for that could be due to the richness in the diversity of experimental approaches in the field of creativity research and the enormous variety in the ways of measuring and interpreting the creativity performance [24,25,26].

According to the latest World Economic Forum report, creativity is one of the most important key skills needed in future jobs [27]. Therefore, in order to help children with their self-realization in the future, education ought to focus on the factors that influence the development of their creativity and contribute to their development.

As mentioned above, multiple studies indicate the positive effects of PA on various cognitive functions. It is also known that creativity is based on various cognitive functions such as cognitive flexibility, inhibitory control, working memory updating, fluency, originality, and insights [28]. However, there is limited research regarding the relationship between PA, motor fitness, and creativity in early-aged school children. There is an even smaller number of studies examining the influence of PA and motor fitness level on the development of creativity, let alone with the mediating role of a gender. Such studies could shed a new light on the most effective strategies for stimulating children’s creativity in a gender perspective, though should also consider longitudinal changes.

It is known that early-aged school pupils are subject to extensive physical changes [29]. During the process of physical growth, speed, endurance, and strength increase in both genders; however, the development pattern of muscle strength occurs more slowly than the development of speed and endurance. Apparently, girls have greater flexibility and coordination than boys at primary school age based on individual performance ranges [29,30]. But Popovici et al. [31] showed that it was boys who had better lower and upper limb strength and abdominal muscles, while girls exceeded in body balance. On the other side, boys achieve significantly higher endurance running performance compared to girls [30].

Regarding the cognitive development of children, research confirms that the cognitive abilities of early school-age children show few differences depending on gender [32,33,34]. In 2011, Ardila et al. [32] carried out research to assess the gender differences in cognition (i.e., verbal, spatial, and arithmetic abilities) in children aged 5 to 16 years. To evaluate cognitive abilities, participants performed the Child Neuropsychological Assessment (CNA) test for seven different cognitive domains (memory, sensory perception, attentional abilities, oral language abilities, metalinguistic awareness, visuospatial, and visuomotor processing). Significant differences in results were observed only in three domains (language, spatial, and sensory-perceptual abilities), and in each of them male participants outperformed female ones. The authors concluded that gender differences could only be found in a small number of the total variance in scores. Therefore, Ardila et al. [32] found only minimal gender differences in cognition during developmental age in children.

In another study, Gur et al. [33] examined a sample of 3500 students aged 8 to 21 who performed the Penn Computerized Battery (Penn CNB) test. The battery consists of 14 single tests and explores many aspects of cognitive functions, such as sensorimotor speed, spatial ability, attention, working, and spatial verbal memory, among others. The results of this study showed that female students, when compared with their male peers, demonstrated higher accuracy and speed on verbal memory, social cognition, and face memory, but they performed worse than males in accuracy and attention speed and spatial and working memory tests. In addition, male students outperformed females in the accuracy and speed of spatial abilities, sensorimotor speed, and locomotor speed tests. Gur et al. [33] emphasized that gender differences become more pronounced after mid-adolescence.

Although the question of whether there are differences in the cognitive abilities of male and female children remains to be studied, the amount of research in this direction is growing [35,36,37,38]. On the other hand, there is a gap in the study of creativity in children depending on gender. It is currently unclear whether gender differentiates creativity, specifically in early school age. The lack of attention paid to gender differences in the creative abilities of males and females in early school age means that we do not know precisely how to influence the development of these abilities and miss the opportunity to effectively influence the development of children’s creativity at school. The next important question is whether gender differences are established in children’s creativity depending on their level of PA and motor fitness. Clarification of these issues could significantly affect the content of the school curriculum in primary school and, in particular, the content of the physical education program.

Therefore, based on the above-mentioned findings and research studies, we have developed a study that aimed at finding out whether there is a gender difference in creativity, PA, motor fitness, and motor creativity in children aged 8–9 years as well as establishing whether there is a relationship between these variables.

## 2. Materials and Methods

### 2.1. Participants 

The study included 195 primary school children (92 girls and 103 boys) aged 8–9 years old from grade 2. Descriptive characteristics of the sample group are presented in Table 1.

All children were recruited from three primary schools and attended a standard school curriculum in the city of Poznan (urban area) in Poland. The research was carried out between January and February 2024. The study was conducted in accordance with the Declaration of Helsinki. The study protocol was approved by the Local Bioethics Committee of The Karol Marcinkowski University of Medical Sciences in Poznan (decision number 400/23). All parents or guardians provided written informed consent.

### 2.2. Study Procedure

The study included assessments of anthropometric data (body height and weight), motor fitness level, self-reported PA, creativity, and motor creativity. All measures and tests were performed in the school setting (gym and classroom). First, a creativity test was conducted in a classroom, and then motor fitness and motor creativity tests were performed in a school gym. The participants were informed about the test techniques and each test item was accompanied by detailed instructions presented to the participants by a trained research assistant. The exclusion criteria for this study were cognitive or physical dysfunction in pupils.

### 2.3. Creativity Assessment

Creativity was determined through the use of the Test for Creative Thinking-Drawing Production (TCT-DP) carried out according to the protocol of Urban [39]. Participants were asked to complete a drawing with six flawed elements placed on a creativity test sheet according to the method of Jellen and Urban [40]; all students performed this test autonomously and by themselves. The examiner noted the end time of the drawing test. Following 15 min from the start of the study, examiners collected sheets from students who had not yet submitted the test. The assessment of the TCT-DP consisted of fourteen criteria, which include the following: (1) continuations, (2) completions, (3) new elements, (4) connections made with a line, (5) connections that contribute to a theme, (6) boundary breaking that is fragment-dependent, (7) boundary breaking that is fragment-independent, (8) perspective, (9) humor and affectivity, (10) unconventionality with, manipulation of the test material, (11) unconventionality with, abstract elements, (12) unconventionality in the use of symbols, (13) unconventionality with unconventional usage of the given fragments, and (14) speed [39]. These creativity scores were assessed by a qualified psychologist. The final TCT-DP result was formed of the sum of the points obtained in all tested criteria.

The reliability and validity of the TCT-DP has been provided in many studies [39,41,42], and it has been previously reported that eventual experience in drawing is unrelated to the TCT-DP score [39]. In the case of Polish standardization studies, internal consistency was estimated based on the Cronbach’s alpha coefficient. The obtained Cronbach alpha coefficients ranged from 0.62 (students of the army cadets’ school) to 0.80 (preschool children) [41].

### 2.4. Motor Creativity Assessment

Motor creativity was evaluated with use of Torrance’s ‘Thinking Creatively in Action and Movement’ (TCAM) test. The administration and the scoring guide of the test were translated back-to-back from English to Polish, and some appropriate adaptations were made by a trained psychologist. The test consists of four activities. The first, third, and fourth activities help to assess motor fluency and originality, whereas the second activity assesses imagination. Fluency is determined as the ability to create alternative modes of movement. Originality is the ability to produce novel, unique, and unusual ways of movement. Imagination is defined as the ability to imagine, fantasize, and assume unusual roles. In the first activity, the participant was asked to overcome the designated distance of three meters in as many ways as possible. The second activity requires the child to imagine themself in six fiction situations and perform appropriate actions accordingly. Four of these situations require the child to pretend to be an animal/object (tree, rabbit, fish, snake), and in the last two the child is expected to pretend and perform some actions (driving a car or pushing an elephant off a desired object). In the third activity, the child is asked to describe or demonstrate as many ways as possible of putting a paper cup into a wastebasket, placed within a two-meter distance. For the fourth activity, the participants are asked to list different/show the potential uses for a paper cup. Testing time ranged between 10 and 30 min. Fluency was projected as a sum of the responses recorded on predetermined score sheets. Originality was assessed (calculated) with the use of the tests’ original reference list of the most frequent responses and points were awarded accordingly. Imagination was assessed on a five-point rating scale (from 1 = no movement to 5 = excellent imitation). All marking of scores were performed accordingly to the original manual [43]. Reliability coefficients for the separate activities were 0.71 for the first activity, 0.79 for the second activity, 0.67 for the third activity, and 0.58 for the fourth activity. The overall reliability coefficient of Torrence TCAM test was 0.84 [43].

### 2.5. Assessments of Anthropometric Parameters

Body height and weight were measured without shoes using an anthropological instrument (Wunder Sa. Bi. Srl., Milan, Italy). Height was measured to the nearest 0.1 cm. Weight was measured to the nearest 0.1 kg. The measurements were performed once by trained research assistants.

### 2.6. Motor Fitness

The motor fitness levels of the participants were assessed using the selected tests from Eurofit battery (Counsil of Europe, Committee for the development of sport, Strasbourg, France) [44]: 10 × 5 m Shuttle Run (SHR)—to determine running speed and agility; 20 m endurance Shuttle Run (Beep test)—to assess cardiorespiratory endurance. 

The 10 × 5 m SHR test involves participants running back and forth over a 5-m distance, changing direction 10 times to measure speed and agility [45].

In the 20 m Shuttle Run test, the participants are asked to stand behind the lines facing the second line (the distance between the lines is 20 m), and then on the special sound signal start the running. The speed at the beginning is quite slow. The participants continue to run between the two lines turning when signaled by recorded beeps. After one minute, a sound indicates an increase in speed, and the beep sound increases its frequency as well. This progress continues each minute (level) to the 15 min level. The participant is given a warning if they fail to reach the line (within 2 m) the first time, and after a second warning they are eliminated by the research assistant. The score is given as a number of levels based on the number of 20 m shuttles reached before the participant was unable to keep up with the recorded beep sound. The final score is the last level completed [46].

The Piórkowski test was used to assess eye–hand coordination, precision of movements, and speed reaction. The Piórkowski test was conducted with the use of a Piórkowski apparatus (AP) (Psychology laboratory “Driver”, Ustrzyki Dolne, Poland). This apparatus has 10 buttons arranged in one row. There is an LED above each button that lights up to indicate the button to press. Only 1 LED always lights up. The task is—using both hands—to press each subsequent button indicated by the device. The device does not wait for the correct press but sets its own pace, and the subject must be able to hit the button correctly as many times as possible. In this study, parameters included a 60 s test with a stimulus presentation frequency of 30 pulses. The outcome was the number of correct responses. A thorough instruction was always provided before the examination. This test has been previously conducted under Polish conditions in the study by Tomczak et al. [47] and Merkisz et al. [48].

### 2.7. Physical Activity Survey

To determine the level of moderate-to-vigorous physical activity (MVPA), the Physical Activity Screening Measure was used [49]. The scale was administered to children on a “one-to-one” basis by a trained staff member of the research team in comfortable conditions for the child. Pupils were asked the following questions: (1) “Over the past 7 days, how many days were you physically active for a total of at least 60 min per day?” and (2) “Over a typical or usual week, how many days are you physically active for a total of at least 60 min per day?” (0 days, 1 day up to 7 days). The final MVPA index score was calculated with the formula provided by Prochaska et al. [49], MVPA = (Q1 + Q2)/2, where Q1 is the number of physically active days over the last 7 days and Q2 is the number of physically active days in a typical week. According to Prochaska et al. [49], the measure is reliable (ICC = 0.77). The item has been proven to have reasonable validity and moderate reliability [50]. This measure has been tested earlier in some Polish studies [51,52,53,54]. 

## 3. Statistical Analysis

Statistical analyses were performed for physical fitness components, PA, and creativity components. After checking for normality with the use of the Shapiro–Wilk test, the lack of normal distribution was noticed; therefore, to compare differences in motor fitness, self-reported PA, creativity, and motor creativity between boys and girls, the Mann–Whitney U test was employed. The correlation between motor fitness, PA, and individual creativity indicators in boys and girls was determined using Spearman’s correlation. For statistical testing, Statistica 13.3 was used (Statsoft, Kraków, Poland), and statistical significance was set at *p* < 0.05.

## 4. Results

A comparison of PA, motor fitness, creativity, and motor creativity test results is presented in Table 2. Regarding motor fitness, boys, as opposed to girls, obtained better results in the 10 × 5 m SHR test (*p* < 0.001) and 20 m Shuttle Run test (*p* < 0.05). In the Piórkowski apparatus test, girls achieved higher (better) results than boys (*p* < 0.05). There was no statistically significant difference between boys and girls in MVPA score, nor were there statistically significant differences in creativity or any of the motor creativity variables between genders.

In boys, the TCT-DP score was not associated with PA (MVPA) and neither with any of the three other indicators of motor fitness. No correlation was noted between motor creativity and PA. Regarding motor fitness and motor creativity, the only correlation, which was mild in power (0.28), was noted between results of the Piórkowski apparatus test and one of the subcategories of creativity, namely originality. No other correlations were noted (Table 3).

In girls, the TCT-DP score also was not associated with PA (MVPA) or with any of the other three indicators of motor fitness. Furthermore, there was no correlation noticed between motor creativity and PA. Regarding motor fitness and motor creativity, a correlation between results of the Piórkowski apparatus test and fluency (one of the TCAM creativity subcategories) was observed, but it was only mild in power (0.24). No other correlations were noted (Table 4).

## 5. Discussion

The findings of the study show some gender differences in creativity, motor creativity, PA, and motor fitness in 8–9-year-old children.

Regarding motor fitness, we confirmed the findings of the previous studies [29,30,31] regarding the existence of differences (with statistical significance) in motor fitness between boys and girls in the 8–9 year old age range. Boys obtained better results in running speed and agility (10 × 5 m SHR test) (*p* < 0.001) as well as cardiorespiratory endurance (20 m Shuttle Run test) (*p* < 0.05) when compared to girls. On the other hand, girls achieved better results than boys in eye–hand coordination, precision of movements, and speed reaction (Piórkowski apparatus test) (*p* < 0.05). Interestingly, no statistically significant difference between genders in PA levels was noticed, indicating that, at least at this age, boys and girls are active to a similar extent.

In terms of creativity and motor creativity in the study, it was found that there was no statistically significant difference between the genders. The results of the TCT-DP showed that there is no statistically significant difference between the two genders in creativity, and this is intersected with the results of previous studies [32,33,34] comparing the other cognitive skills of boys and girls in early school age. In the study of Ardila et al. [32], a sample of monolingual children from Mexico and Columbia (350 boys, 438 girls, age range 5–16 years) were examined, and it was observed that the gender differences in cognitive development were minimal and appeared only in a small number of tests, which only contributed a low percentage to the total difference. In turn, in the study of Gur et al. [33], the gender effect on cognitive skills was noted, but it was much smaller than the effect of age. It is worth mentioning that they tested youths ages 8–21 years, and testing included performance accuracy and response times for executive control, episodic memory, complex cognition, social cognition, and sensorimotor speed domains. In that sample, gender differences (in most domains of tested items) were already seen at the youngest age groups, but it was with age that the discrepancy became much more noticeable. The majority of those gender differences were apparent by early adolescence [33].

In our study, the TCAM showed similar results regarding all variables of motor creativity—boys and girls aged 8–9 years did not differ by this indicator either. Earlier, similar findings were obtained by Zachopoulou and Makri [55] in their study that investigated the motor creativity of 191 young children aged 5–8 years. In their study, the subject’s motor creativity was assessed using the Divergent Movement Ability Test, where the effect of gender in two factors of motor creativity—motor fluency and motor flexibility—was estimated. No statistically significant differences between the two genders were found [55]. Thus, one can conclude that early-aged young girls and boys have similar levels in terms of motor creativity performance.

According to the correlation between the various variables of our study, the following was established. In both boys and girls, creativity was not associated with PA. There was also no correlation between motor creativity and PA for either gender. It seems that in 8–9-year age range for both gender, creativity does not correlate with PA. Results obtained by Piya-amornphan et al. [56], who investigated the relationship between creativity and PA among 1447 students with different age groups (from 6 to 17 years) in 34 schools from southern Thailand, are in line with our study. The authors, in their study, also used the Test for Creative Thinking-Drawing Production (TCT-DP) to measure creativity. PA was determined with the use of the Thailand Physical Activity Children Survey-the Student Questionnaire (TPACS-SQ). The results of their research showed that in children aged 6–9 years and 10–13 years, creativity was not associated with PA in either of the age categories. The correlation between creativity and PA was observed only in participants aged 14–17 years [56], indicating the possible longitudinal developmental factor.

A growing body of research [6,14,15,57] demonstrates that children’s participation in PA has a positive effect on cognitive function and academic achievements in school through positive effects on general brain structure and functioning and, thus, on cognition [58]. But it seems that the mechanism for creative development is different. Based on established data, which show that PA has a positive effect on brain functions [57] and that youth aged 14–17 with a higher level of activity had a higher level of creativity, it can be assumed that PA gives children a predisposition to develop creativity, but the stimulation of creative development occurs due to additional influencing factors. Perhaps, this is why older children, who had more time opportunities for additional influence on the development of creativity, including higher PA as an extra factor, had a higher level of creativity at the end. In this case, it would imply the auxiliary function of PA in the development of creativity, although it may not affect this process directly. Therefore, it can be assumed that the development of creativity requires special methods and development strategies.

Another possible explanation of this thesis might be the assumption that engagement in various movement activities not only has a direct effect on the development of creativity but also has an accumulative effect and requires a longer exposure time. This could also explain why the connection between PA and creativity is not observed in children aged 6–13 years, but it is observed in older children. This question needs further, more in-depth research, which should examine the relationship between creativity and previous experiences.

With regard to motor fitness and creativity, in our study, in both boys and girls, creativity did not correlate with measured indicators of motor fitness. In the case of motor fitness and motor creativity, only a weak correlation was noted between the results of the Piórkowski test and the subfactor of motor creativity, namely originality for boys (r = 0.28) and the Piórkowski test and fluency for girls (r = 0.24). The results potentially suggest that motor proficiency and creativity may not be inter-related traits at this early age of school children. Marinšek and Lukman [59], in their study of the relationship between motor creativity and motor fitness, found similar results. The authors used the Test of Gross Motor Development and Bertsch’s motor creativity test among 5–6 years old children for the evaluation of motor skill proficiency and motor creativity. No correlations between motor skill proficiency and motor creativity were found in the examined group of children. In their study, the authors concluded that motor creativity is a trait that is independent from motor skill proficiency in early-age children, and one cannot and should not predict motor creativity basing on the knowledge of a child’s level of motor skill development in this age category [59]. These results are also in line with previous work from Scibinetti et al. [60].

It seems that physical education classes, based mainly on the reproduction of existing motor skills and physical drills, do not contribute to the development of motor creativity. They affect motor skills and motor fitness, mostly with linear progression (from easier to more complex motor skills), taking into account the efficiency of movements. However, motor creativity mainly arises from activities that facilitate non-linear mechanisms and largely ignore the efficiency of movement. Typically, in linear mechanisms, the constraints of the task are very narrow. They do not allow children to explore new possibilities of movement [59,61,62]. Based on this, it can be assumed that the development of motor creativity requires a specially designed teaching strategy.

Using objective methods of measuring motor fitness and creativity can be considered a strength of our study, but there are some limitations as well. First, the sample’s age range was narrowed to children aged 8–9 years, so, generalization, especially given that the motor development process during this period is very dynamic, is limited [29]. Second, all data on children’s PA were self-reported, which to some extent depends on recall, and reporting bias is possible. Third, the research design limits us to establishing casual relationships between PA, motor fitness, creativity, and motor creativity; therefore, future studies should use samples of other age categories or a controlled experimental design with modulations of creativity intervention programs. Furthermore, longitudinal studies would be of value in these aspects.

## 6. Conclusions

The results of this research indicate some significance differences in motor fitness levels between boys and girls aged 8–9 years but a lack of such differences in PA, creativity, and motor creativity levels. Moreover, no correlation between PA, motor fitness, and creativity nor motor creativity were noticed in either of the genders. Thus, it can be pointed out that in order to develop children’s creativity through PA, it is necessary to create an appropriate environment and strategy that will strengthen, inspire, and promote children’s creativity through movement.

Creativity is one of the most demanded work skills in today’s labor market and education should be held responsible for providing such opportunities to school pupils. The development of creativeness can be stimulated via various school subjects—generally the better pedagogically prepared is the teacher, the more educational chances they can provide. However, physical education, with its variety of contexts, sports, and wide range of teaching methods, probably seems to be the best platform. Therefore, knowing there is no difference in creativity between the genders in early-aged school children, we need to search for the new, more effective teaching/learning method, which would effectively awaken that kind of skills in co-educational settings.

## Figures and Tables

**Table 1:** Descriptive characteristics.

	Boys	Girls
Variables	M ± SD	M ± SD
Gender	103 (52.82)	92 (47.18)
Age (years)	8.01 ± 0.33	8.01 ± 0.23
Body height (cm)	134.07 ± 6.43	131.61 ± 6.15
Body mass (kg)	31.30 ± 7.35	29.32 ± 5.64

Notes: M—mean; SD—standard deviation; cm—centimeters; kg—kilograms.

**Table 2:** A comparison of the motor fitness, PA, creativity, and motor creativity test scores between boys and girls.

	Boys	Girls	*p*
Variables	M ± SD	M ± SD	
10 × 5 m SHR (s)	24.3 ± 2.69	25.9 ± 2.06	<0.001
20 m Shuttle Run (lvl)	3.3 ± 1.33	2.9 ± 0.96	<0.05
Piórkowski apparatus (no/30 pulses)	23.6 ± 4.61	24.8 ± 3.99	<0.05
MVPA (hrs/week)	4.1 ± 1.58	3.8 ± 1.23	>0.05
TCT-DP (pts)	22.7 ± 9.93	24.2 ± 10.07	>0.05
TCAM Fluency (pts)	24.5 ± 9.24	22.6 ± 9.79	>0.05
TCAM Originality (pts)	26.3 ± 12.58	25.5 ± 13.82	>0.05
TCAM Imagination (pts)	18.7 ± 5.42	18.9 ± 4.61	>0.05

Notes: M—mean; SD—standard deviation; no—number; s—seconds; lvl—level; pts—points; SHR—10 × 5 m shuttle run; MVPA—moderate-to-vigorous PA; TCAM—Torrance’s ‘Thinking Creatively in Action and Movement’ test; TCT-DP—Test for Creative Thinking-Drawing Production.

**Table 3:** The correlation between motor fitness, PA, and individual creativity indicators in boys.

Variables	TCT-DP	TCAM Fluency	TCAM Originality	TCAM Imagination
10 × 5 m SHR	−0.17	0.01	0.02	−0.05
20 m Shuttle Run	0.13	−0.03	−0.13	0.06
Piórkowski apparatus	0.04	0.12	0.28 *	0.13
MVPA	0.01	−0.07	0.01	0.08

Notes: SHR—10 × 5 m shuttle run; MVPA—moderate-to-vigorous PA; TCAM—Torrance’s ‘Thinking Creatively in Action and Movement’ test; TCT-DP—Test for Creative Thinking-Drawing Production; * *p*-value ≤ 0.05.

**Table 4:** Correlation between motor fitness, PA and individual creativity indicators in girls.

Variables	TCT-DP	TCAM Fluency	TCAM Originality	TCAM Imagination
10 × 5 m SHR	−0.03	0.06	0.15	0.03
20 m Shuttle Run	0.02	0.09	−0.08	0.06
Piórkowski apparatus	0.04	0.24 *	0.17	0.15
MVPA	−0.01	−0.01	0.01	0.02

Notes: SHR—10 × 5 m shuttle run; MVPA—moderate-to-vigorous PA, TCAM—Torrance’s ‘Thinking Creatively in Action and Movement’ test; TCT-DP—Test for Creative Thinking-Drawing Production; * *p*-value ≤ 0.05.

## Data Availability

The datasets used and/or analyzed during the current study are available from the corresponding author upon reasonable request due to privacy.
